# Multi-position data collection and dynamic beam sizing: recent improvements to the automatic data-collection algorithms on MASSIF-1

**DOI:** 10.1107/S2059798318003728

**Published:** 2018-04-24

**Authors:** Olof Svensson, Maciej Gilski, Didier Nurizzo, Matthew W. Bowler

**Affiliations:** a European Synchrotron Radiation Facility, 71 Avenue des Martyrs, CS 40220, 38043 Grenoble, France; b European Molecular Biology Laboratory, Grenoble Outstation, 71 Avenue des Martyrs, CS 90181, 38042 Grenoble, France

**Keywords:** X-ray centring, synchrotron instrumentation, macromolecular crystallography, automation, helical data collection, multiple-crystal data collection, MASSIF-1, data-collection algorithms, robotics

## Abstract

Macromolecular crystallography is now a mature and widely used technique that is essential in the understanding of biology and medicine. Increases in computing power combined with robotics have not only enabled large numbers of samples to be screened and characterized but have also enabled better decisions to be taken on data collection itself. This led to the development of MASSIF-1 at the ESRF, the first beamline in the world to run fully automatically while making intelligent decisions taking user requirements into account. Since opening in late 2014, the beamline has processed over 42 000 samples. Improvements have been made to the speed of the sample-handling robotics and error management within the software routines. The workflows initially put into place, while highly innovative at the time, have been expanded to include increased complexity and additional intelligence using the information gathered during characterization; this includes adapting the beam diameter dynamically to match the diffraction volume within the crystal. Complex multi-position and multi-crystal data collections have now also been integrated into the selection of experiments available. This has led to increased data quality and throughput, allowing even the most challenging samples to be treated automatically.

## Abstract 2

Significant improvements to the sample-location, characterization and data-collection algorithms on the autonomous ESRF beamline MASSIF-1 are described. The workflows now include dynamic beam-diameter adjustment and multi-position and multi-crystal data collections.

## 1. Introduction   

Automation is transforming the way that scientific data are collected, allowing large amounts of high-quality data to be gathered in a consistent manner (Quintana & Plätzer, 2015 ▸; Foster, 2005 ▸). Advances in robotics and software have been key to these developments and have had a particular impact on structural biology, allowing multiple constructs to be screened and purified (Camper & Viola, 2009 ▸; Hart & Waldo, 2013 ▸; Vijayachandran *et al.*, 2011 ▸); huge numbers of crystallization experiments to be performed (Elsliger *et al.*, 2010 ▸; Ferrer *et al.*, 2013 ▸; Heinemann *et al.*, 2003 ▸; Joachimiak, 2009 ▸; Calero *et al.*, 2014 ▸); samples to be mounted at synchrotrons (Cipriani *et al.*, 2006 ▸; Cohen *et al.*, 2002 ▸; Jacquamet *et al.*, 2009 ▸; Nurizzo *et al.*, 2016 ▸; Papp *et al.*, 2017 ▸; Snell *et al.*, 2004 ▸); data to be analysed and processed (Bourenkov & Popov, 2010 ▸; Holton & Alber, 2004 ▸; Incardona *et al.*, 2009 ▸; Leslie *et al.*, 2002 ▸; Monaco *et al.*, 2013 ▸; Winter, 2010 ▸); and the entire PDB to be validated (Joosten *et al.*, 2012 ▸). The combination of robotic sample mounting and online data analysis has been particularly important in macromolecular crystallography (MX) as it has allowed time to be saved, large numbers of samples to be screened, and enabled the remote operation of beamlines. However, despite these advances, a human presence is still required to sequence actions. Pioneering beamlines that have fully automated the process, such as LRL-CAT at the Advanced Photon Source (Wasserman *et al.*, 2012 ▸) and the Stanford Synchrotron Radiation Lightsource MX beamlines (Tsai *et al.*, 2013 ▸), removed the need for a human presence, but as they rely on optical loop centring this means that restrictions have to be placed on the size of the crystals and they tend to be robust, well diffracting samples, generally those for proprietary research in the pharmaceutical industry.

In 2014 the ESRF beamline MASSIF-1 (Bowler *et al.*, 2015 ▸) opened to users as the first beamline to fully automate MX data collection, including sample-location and complex decision-making algorithms (Svensson *et al.*, 2015 ▸). The combination of sample centring using X-ray diffraction quality as a metric (X-ray centring) and characterization allows even the smallest and most weakly diffracting samples to be treated automatically. This opened full automation to any sample presented in any mount and has provided a new tool to structural biologists, allowing the process of collecting hundreds of data sets or screening hundreds of crystals to be ‘outsourced’, freeing their time and often leading to the collection of better data (Bowler *et al.*, 2016 ▸). At the time of writing, the beamline has processed more than 42 000 samples representing a wide range of projects, from those that require extensive screening to find the best diffracting crystal (Na *et al.*, 2017 ▸; Sorigué *et al.*, 2017 ▸; Naschberger *et al.* 2017 ▸) to small-molecule fragment screening (Cheeseman *et al.*, 2017 ▸; Hiruma *et al.*, 2017 ▸) and experimental phasing at high and low resolutions (Kharde *et al.*, 2015 ▸; Muir *et al.*, 2016 ▸; Sabbadin *et al.*, 2018 ▸). The beamline is able to deal with a wide range of samples by combining parameters provided by the user with information gathered during processing. The workflows that were initially put into place have performed very well, but many enhancements remained possible.

Here, we describe how the hardware and algorithms have been improved to increase the amount and the quality of data collected on MASSIF-1. The speed of the robotics has been increased and additional subroutines have been added to monitor and correct errors in centring and to account for low-resolution data collection as well as dynamically adjusting the beam diameter to match homogenous diffraction volumes. In combination with new multiple-position and multiple-crystal data-collection workflows, fully automatic data collection is now possible for the most challenging samples.

## 2. Experimental details and results   

### 2.1. Hardware improvements   

**Figure 1:**
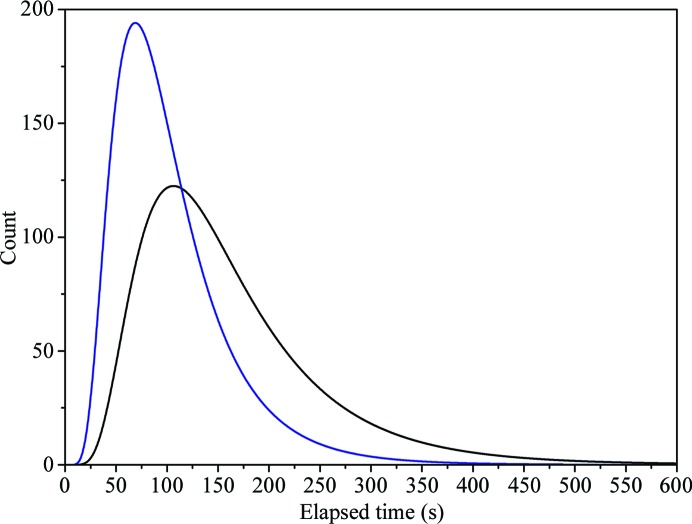
The decrease in the time taken to perform the mesh scan after hardware improvements. Log-normal distribution of the elapsed time for mesh scans using the former protocol (black) and the new fast mesh (blue) for the two months preceding and following the implementation of the new protocol.

One of the most time-consuming, and important, steps in the X-ray centring process is the initial mesh scan that locates and characterizes the crystal. When first implemented on MASSIF-1, a rotation of ω was included in the scan in order to avoid still images that can cause problems in processing. This implementation required that triggering of the acquisition of images was instigated by the ω axis, meaning that each line of the mesh was treated as a separate data collection. The preparation required between data collections led to additional time being taken for the scan. We have now implemented a scan that includes no rotation of the ω axis and only requires movements of the high-precision *Y*/*Z* stage beneath the RoboDiff (Nurizzo *et al.*, 2016 ▸). This allows the triggering of acquisition to be made by the motor position, allowing the whole mesh to be launched as a single data collection (Aishima *et al.*, 2010 ▸). This method was implemented in 2017, and analysis of data for two-month periods before and after its implementation showed that the time required for these scans has been reduced by an average of 1 min (Fig. 1 ▸).

### 2.2. Dynamic beam sizing   

**Figure 2:**
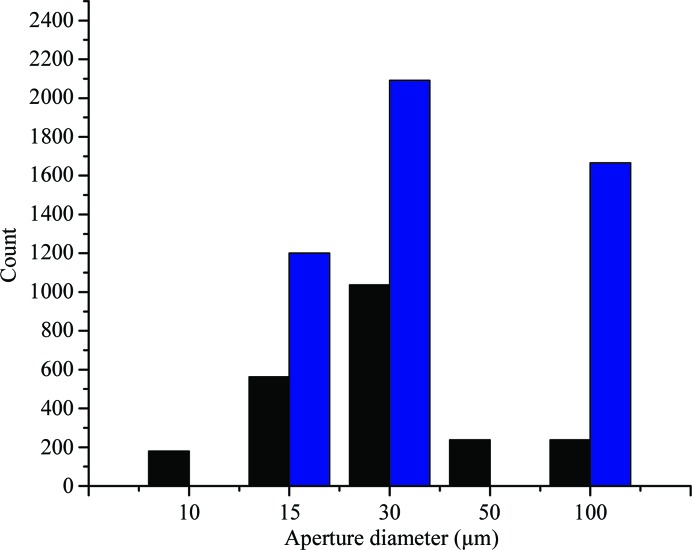
Beam-diameter selections by users and the algorithm in 2017. The number of times a beam diameter was selected either by the user (black) or automatically (blue) is shown. 6877 data collections were performed with the beam diameter at the default value of 50 µm; as this is not changed by the software, the value is not shown.

One of the benefits of running a completely automated system is the ability to collect large amounts of data from samples and to use these data to improve strategies for data collection. We initially realized that the volumes of all crystals were determined during the X-ray centring routine, and this information was subsequently included in the strategy calculation, having the largest effect on the calculation of the maximum dose given to a crystal during data collection (Bowler *et al.*, 2016 ▸; Svensson *et al.*, 2015 ▸). Additionally, these measurements provided a distribution of crystal volumes, allowing us to use a default beam diameter of 50 µm, as this was the crystal dimension that was most frequently observed on the beamline. A specific beam diameter can be selected on a per-sample basis in the diffraction plan in ISPyB (Delagenière *et al.*, 2011 ▸); however, this option is usually used when users are sure that the crystal volumes are significantly smaller than the default beam diameter (Fig. 2 ▸). Using the information gathered during the mesh scan, we can determine an optimized beam diameter. By accurately matching the beam diameter to the crystal, it has been shown that the background can be dramatically reduced (Moukhametzianov *et al.*, 2008 ▸). This is particularly striking when the crystals are very small (Evans *et al.*, 2011 ▸), but if a crystal is large the additional diffraction power should not be wasted. This also has to be balanced with the degree of variability within each crystal (Bowler & Bowler, 2014 ▸; Bowler *et al.*, 2010 ▸; Pozharski, 2012 ▸).

We have now introduced a dynamic beam-diameter adjustment into all workflows running on MASSIF-1 where no value has been pre-selected by the user. The X-ray centring routine determines the crystal position relative to the beam and the crystal dimensions, and also determines the best homogenous volume within the crystal. The centre of mass of this volume is then used as the centring position and it is the dimensions of this volume that are used to select the beam diameter. There are five beam diameters available on MASSIF-1, 100, 50, 30, 15 and 10 µm (Bowler *et al.*, 2015 ▸), and the smallest vertical volume dimension is used to select the aperture that matches most closely. In this way, the largest volume can be illuminated without increasing the background or ‘contaminating’ the diffraction from variable areas. All steps during X-ray centring are performed with the 50 µm aperture. As the scans are performed with an overlap, the smallest dimension that can be measured is ∼20 µm, meaning that the 10 µm option is only used when selected by the user. Once X-ray centring is completed, the new aperture diameter is selected and characterization images are collected using this diameter. The strategy calculation will include the new flux for the aperture as well as the crystal volume determined during the centring procedure. Since introducing the adaptable beam diameter the system has selected the 30 µm beam size most frequently (Fig. 2 ▸), followed by the 100 and 15 µm diameter apertures. Half of all data collections are performed with a diameter of 50 µm, reinforcing its choice as the default value.

Can the advantages of dynamic beam adjustment be demonstrated in a consistent manner? It is always problematic to clearly show that one data-collection method is better than another. However, here we show that dynamic beam sizing makes a significant difference for weakly diffracting samples.

We initially tested the adaptive beam-diameter protocol on crystals of the β_1_-adrenergic GPCR (Warne *et al.*, 2008 ▸). These crystals diffract weakly, exhibit considerable variation in diffraction quality and tend to form as thin plates or needles. A total of 30 crystals were run on MASSIF-1, first using the classic protocol (Svensson *et al.*, 2015 ▸), where a 50 µm beam diameter is the default, and then running a second protocol on the same crystal including the adaptive beam diameter. In many cases data sets were collected from the same crystal using both procedures; however, for some cases data sets were only processed where the beam diameter had been reduced to match the crystal size. Table 1 ▸ shows crystal dimensions and data-processing statistics for crystals where an automatically processed data set was produced (eight out of 30 crystals). Where crystals were of sufficient quality, data sets were mostly produced using both protocols, but it is for the smaller crystals that a difference is discernible. For crystals with a *y* dimension below 30 µm the data sets produced have a higher 〈*I*/σ(*I*)〉 or resolution limit (Table 1 ▸; adrcpt-For42, adrcpt-For48, adrcpt-For59 and adrcpt-For67) even though the crystals had already been exposed. For one of these crystals, adrcpt-For42, the data set collected with the smaller beam is significantly better (Table 2 ▸).

**Figure 3:**
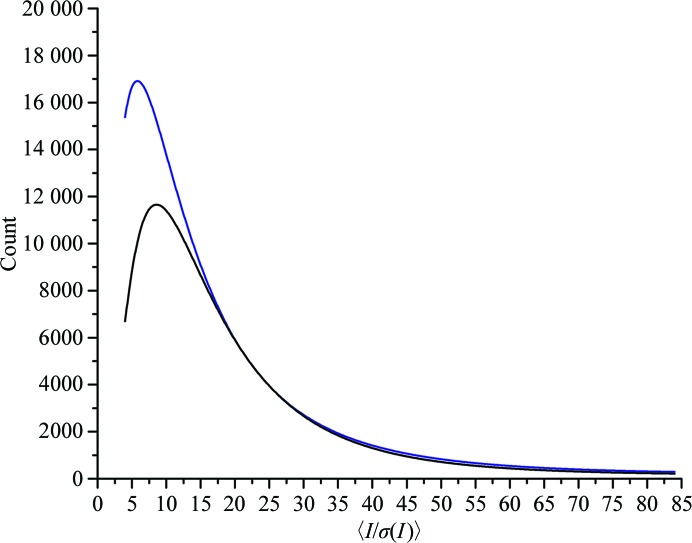
Distribution of overall 〈*I*/σ(*I*)〉 for data sets processed in the year preceding implementation of the dynamic beam aperture (black) and the year after implementation of the dynamic beam aperture (blue). There was a significant shift in the number of data sets processed with lower 〈*I*/σ(*I*)〉 after dynamic beam-diameter adjustment was introduced.

What effect has the protocol had on overall data collection? In order to analyse the difference, we looked at the average signal-to-noise ratios, 〈*I*/σ(*I*)〉, for all data sets processed automatically (Monaco *et al.*, 2013 ▸; Vonrhein *et al.*, 2011 ▸; Sparta *et al.*, 2016 ▸; Winter, 2010 ▸) in the year preceding and following the introduction of the protocol. This amounts to data for approximately 22 000 samples. Fig. 3 ▸ shows the distributions of overall 〈*I*/σ(*I*)〉 for the data sets. While the distributions are similar for high 〈*I*/σ(*I*)〉 they diverge at lower values, with a significant shift lower for the adaptive beam-diameter data sets. The average before the procedure was put in place was 14.4 and it decreased to 12.2 after, with modal values of 8.55 before and 5.9 after (Fig. 3 ▸). We initially found this surprising as we had expected a general increase in 〈*I*/σ(*I*)〉. However, given the effect seen on the GPCR crystals, the distribution change is understandable. While the beam diameter is increased or decreased to match the diffraction volume, the 〈*I*/σ(*I*)〉 values for strongly diffracting crystals will remain the same given the dose to achieve a certain resolution without radiation damage, taking the crystal volume and changed flux into account. However, it was for the weakly diffracting crystals that the adaptive beam diameter had the most significant effect. Whether the diameter was increased or decreased, there was a large shift in the number of data sets that were processed that had rather low 〈*I*/σ(*I*)〉 after the adaptive beam-diameter protocol was implemented. This implies that by introducing this routine into the regular data-collection workflow, the beamline is able to increase the number of data sets processed from these samples by reducing the background noise.

### 2.3. Improved error handling   

The correct handling of errors is paramount in an automated system. We initially introduced many error-handling routines at both a high level, such as the collection of a data set with default settings when indexing fails (Svensson *et al.*, 2015 ▸), and a low level, such as escaping from small robotic errors (Nurizzo *et al.*, 2016 ▸). After processing more than 42 000 samples we have now been able to observe most errors that may be encountered and have extended the processes to catch them.

#### 2.3.1. Centring errors   

We have occasionally observed that after the X-ray centring routine the crystal was still not correctly aligned over the full rotation range. This may be due to movements of the support after the routine has completed or to errors in the routine arising from multiple peaks being selected for centring. Whatever the reason, it can lead to a data set being lost. We therefore introduced a check in the characterization step that ensures that the four images have a diffraction signal. If one image has a signal that is below 10% of the top signal, a recovery routine is launched. This involves three short line scans (50 µm above and below the current centred position) being launched over the currently centred position. In most cases it corrects the error. In 2017, 13 776 samples were processed on MASSIF-1 and centring recovery was launched 221 times. This represents a centring-error rate of 1.6%, which should be reduced further by being able to detect and recover incorrectly centred samples.

#### 2.3.2. Low-resolution data collection   

Calculating an optimized strategy for low-resolution (>4 Å) data collection can be problematic, but is very important as useful information can be extracted from carefully collected data at these resolutions. Here, we used information from low-resolution data collections that had already been performed to improve how the routines deal with these samples. Unless a resolution is specified by the user, all mesh and characterization images are collected at 2 Å. If the predicted resolution extends beyond the corners of the detector (1.42 Å) the detector is moved to the new resolution and a further characterization is launched. This allows the highest possible resolution to be obtained and ensures that characterization is performed at an optimal detector distance (Svensson *et al.*, 2015 ▸). However, for low resolution, the resolution determined by *BEST* is used and data collection proceeds according to the determined strategy. It would seem sensible that if very low diffraction resolution is determined, the characterization images should also be re-collected at this resolution. We therefore introduced a routine to re-collect the characterization images at 4 Å resolution for all samples where the determined resolution is below this value. This allows the distribution of intensity to be better estimated and should lead to better strategy calculations (Popov & Bourenkov, 2003 ▸).

**Figure 4:**
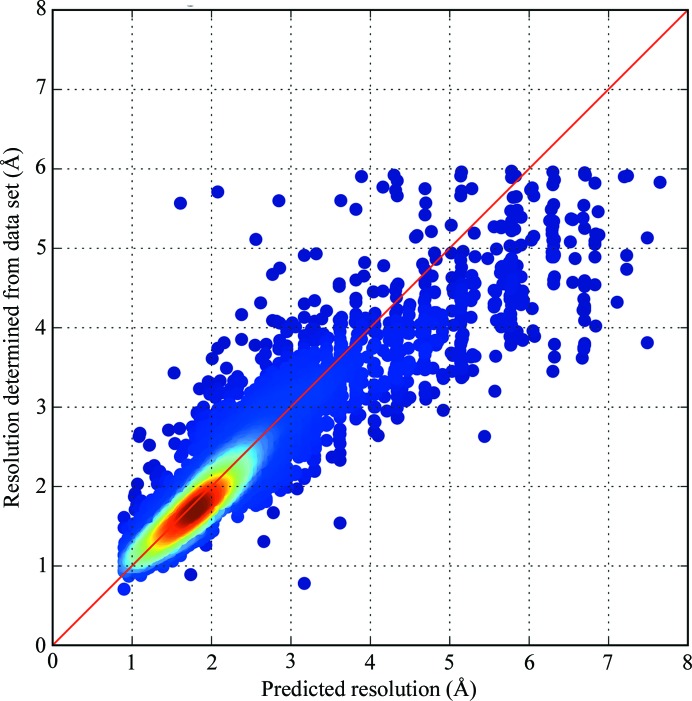
Scatter plot of predicted resolutions for data collections against the resolutions determined by autoprocessing for all crystals processed so far on MASSIF-1 that resulted in a strategy and an automatically processed data set. The red line shows perfect agreement between predicted and achieved resolution and the gradient shows the density of data points.

By analysing the relationship between the predicted and the determined resolution from all data sets collected so far we can also try to improve the quality of the data collected on MASSIF-1 (Fig. 4 ▸). The distribution shows that the agreement is excellent, usually slightly underestimating the achievable resolution. This may well be due to the difference in the criterion for resolution-limit determination, which is 〈*I*/σ(*I*)〉 for characterization and CC_1/2_ for complete data sets. A clear trend is that the strategy tends to underestimate the resolution for weakly diffracting crystals (Fig. 4 ▸). This is due to the difficulty in estimating a *B* factor at very low resolution. In addition to re-collecting the characterization images, the procedure now always sets the detector resolution to 4 Å for all data collections where the predicted resolution is lower than this value. In this way, we hope that higher resolution data will not be missed, if possible, as complete data to 4 Å resolution are more important than suboptimal data collection at 7 Å resolution.

### 2.4. Multiple-crystal and multiple-position data-collection strategies   

**Figure 5:**
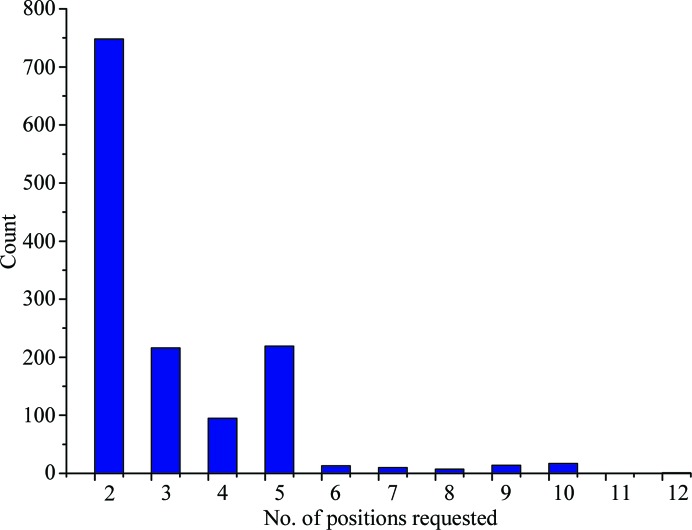
Number of positions selected by users for multiple-crystal and multiple-position data collections in 2017. Multiple-position data collections were requested for 9% of samples.

The possibility to input a number of positions from which to collect data was introduced into the diffraction plan early in the operation of MASSIF-1 (Bowler *et al.*, 2016 ▸; Svensson *et al.*, 2015 ▸). This has allowed complete data sets to be collected either from separate crystals contained on a sample support or from multiple positions within a single crystal and has proved to be a popular option, with many samples received on MASSIF-1 having between two and 12 positions requested (Fig. 5 ▸). While extremely useful, this protocol does not cover the scenario where radiation-sensitive samples can benefit from a large dose being spread over multiple partial data sets, a procedure known generally as helical data collection (Flot *et al.*, 2010 ▸) and that has been shown to be beneficial in many cases (Polsinelli *et al.*, 2017 ▸). Radiation damage can often make it difficult to collect complete data, or data with sufficient anomalous signal, from a single crystal or a single position within a crystal. A new experiment type is now available on MASSIF-1 that will automatically collect multiple partial data sets from positions within a homogenous volume of a crystal. This can lead to improved data quality, increased resolution and higher anomalous peaks. This is the first fully automated helical data-collection protocol that also accounts for the heterogeneity of crystal diffraction quality.

**Figure 6:**
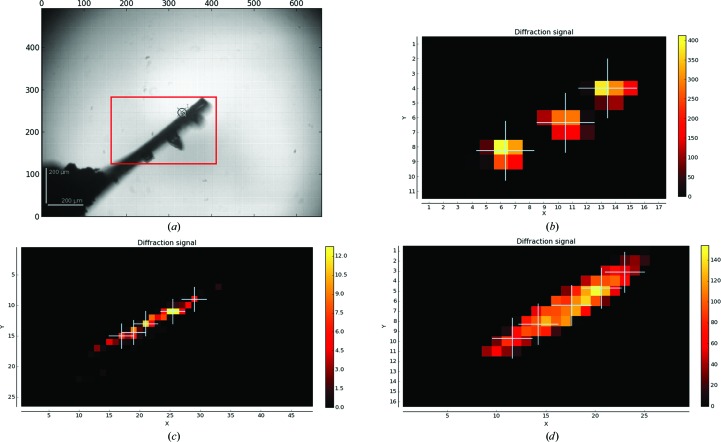
Multiple-crystal and multiple-position data collection. (*a*) *automesh* scan of a CrystalDirect (Zander, Hoffmann *et al.*, 2016 ▸) support that has three crystals mounted. The widest orientation of the mount was selected. (*b*) Mesh scan of the mount shown in (*a*). Three positions were requested and three were detected. (*c*) Mesh scan for a βPGM crystal where a native pseudohelical data collection was requested, five positions were detected and a beam diameter of 30 µm was selected. (*d*) Mesh scan for an FAE crystal where a SAD pseudohelical data collection was requested, five positions were detected and a beam diameter of 100 µm was selected.

Multiple-position/crystal experiments are selected either by specifying a number of positions in the diffraction plan for the sample or by requesting MXPressP or MXPressP_SAD (for a pseudohelical strategy or a pseudohelical with SAD strategy, respectively) in the ‘experiment type’ field for the required samples in ISPyB (Delagenière *et al.*, 2011 ▸). These new experiments operate in much the same way as the usual automated workflows on MASSIF-1 in that all of the current features are retained, such as resolution selection, strategy input, diffraction-volume calculation and smart beam sizing. If multiple positions are selected, the *automesh* algorithm, which determines the area to scan to locate the crystal (Svensson *et al.*, 2015 ▸), uses the widest orientation of the sample support, rather than the smallest, in order to avoid overlapping crystals or positions in ω (Fig. 6*a* ▸). After the mesh scan is complete, the map is analysed either for the number of peaks requested or, if no positions are specified for MXPressP, a default value of five. In the original procedure, a diffraction volume was selected by applying a threshold (50% of the maximum) to the diffraction signal and determining a centre of mass over connected regions (Svensson *et al.*, 2015 ▸). For multi-position experiments the same thresholding is applied but is iterated over the whole crystal volume using the next highest point to start after each volume has been defined (Fig. 6 ▸). The determined volumes must be within 10% of the value of the peak for multiple-crystal data collection or 70% of the second highest peak for pseudohelical data collection. Additionally, any peaks that are closer than a beam diameter together or that will overlap in ω are eliminated. The number of allowed detected volumes is then specified by a comment in ISPyB. If multiple crystals have been selected, each point is then centred as usual and a complete data set is collected according to user input requirements. If MXPressP is selected, the top volume is centred and four characterization images are collected. A strategy is then calculated for a complete data set and the data are collected; for MXPressP_SAD the strategy is calculated for structure solution by SAD (Svensson *et al.*, 2015 ▸). As usual, in the case of indexing failure a default data collection of 180° is collected (240° for triclinic and 360° for SAD data collection). Once completed, a strategy is then calculated to collect a complete data set from the *N* positions determined in the mesh scan that are within 70% of the value of position 2. The strategy, as usual, accounts for the volume of the positions, beam diameter *etc*. Again, in the case of a failure in indexing default data collections are performed at each position using a full dose and a rotation range determined by 180°/*N* (240°/*N* for triclinic and 360°/*N* for SAD). Each partial data set has a 5° overlap with the next to assist with scaling.

We are, for the moment, remaining cautious with pseudohelical data collection by collecting a full single-position complete data set from the best volume. The reason for this is twofold: (i) we have observed that crystal heterogeneity can often lead to a number of the partial data sets being of varying quality despite the stringent quality threshold that we have implemented and (ii) we are eager to compile a large amount of data on how and when helical data collection is superior to single-position data collection. This is extremely important as, so far, the few studies on helical data collection have not considered crystal heterogeneity (Bowler & Bowler, 2014 ▸; Bowler *et al.*, 2010 ▸). Strategy parameters and data-processing statistics for two example systems using the pseudohelical routines for native and SAD data collections are shown in Table 3 ▸. Two proteins that tend to form crystals with a needle morphology were selected: β-phosphoglucomutase (βPGM) in an open conformation (Baxter *et al.*, 2010 ▸) (Fig. 6*c* ▸) and ferulic acid esterase (FAE, Fig. 6*d* ▸), which contains eight Se atoms and five Cd^2+^ ions (Prates *et al.*, 2001 ▸) with a significant anomalous signal at the MASSIF-1 wavelength of 0.966 Å. Comparing the single-position data collection with the merged multiple-position data sets shows that in these cases there is not a significant increase in data quality. However, in the SAD case the helical data set has considerably higher 〈*I*/σ(*I*)〉, anomalous correlation co­efficients and mid-slope of anomalous probability than the single-position data set. For the native data sets, the single position is slightly better. This may reflect the heterogeneity within the crystal and highlights the importance of this parameter in whether to select helical *versus* single position for a certain project. The ability to automatically run clustering algorithms (Giordano *et al.*, 2012 ▸; Zander, Cianci *et al.*, 2016 ▸) on these partial data sets may also improve the quality of the final data. We hope that by being able to analyse the variation in diffraction quality, and compare single-position data with multi-position data from the same crystal, a more general strategy for these types of data collection may emerge.

## 3. Discussion   

The results presented here demonstrate not only the increase in the speed and reliability of automatic data collections but also that more complex strategies can be brought into the arena of autonomous experiments. Automation is often seen as a way to deal with mundane experiments that require little human input. The autonomous system presented here is different in that in addition to automating mounting and centring, it also uses data gathered during the process to improve data-collection strategies. We have already demonstrated that MASSIF-1 collects, on average, better quality data than humans are able to (Bowler *et al.*, 2016 ▸). The additional routines presented here add even more expert knowledge into the system that should further enhance its ability to extract the best possible data from every sample. This built-in expert knowledge means that the system is excellent not only for robust and routine data collections but also for challenging systems that diffract weakly. We have demonstrated that adapting the beam diameter can increase the number of data sets that can be processed from these types of sample. We hope that by providing more data on more samples we can improve feedback into experiment cycles and increase the amount of useful data produced.

All of the developments described here have been exported to the human-operated ESRF beamlines (Mueller-Dieckmann *et al.*, 2015 ▸). As structural biologists now turn to an ever wider variety of tech­niques, we hope that fully automatic data collection will become the standard data-collection method for MX as the best possible data can be collected from samples, be they large and robust or small and weakly diffracting. In combination with developments in the robotic mounting and soaking of crystals (Zander, Hoffmann *et al.*, 2016 ▸), we envision that the future of macromolecular crystallo­graphy is the provision of a fully automated high-throughput service that is able to rapidly produce high-quality structural models and to screen for potential therapeutic and probe molecules.

## Figures and Tables

**Table 1:** Data-collection details for a β_1_-andrenergic GPCR crystal using standard and adapted beam-diameter protocols The dimensions are *x*, the measured crystal length parallel to the spindle axis, *y*, the height orthogonal to the spindle axis, and *z*, the depth orthogonal to the spindle axis 90° away in ω.

		Fixed beam diameter		Adaptable beam diameter	
Crystal	Crystal dimensions (*x* × *y* × *z*) (mm)	Resolution limit (Å)	〈*I*/σ(*I*)〉	Resolution limit (Å)	〈*I*/σ(*I*)〉
adrcpt-For41	0.109 × 0.053 × 0.025	3.77	6.7	4.13	4.4
adrcpt-For42	0.084 × 0.025 × 0.025	4.22	4.3	3.53	10.6
adrcpt-For45	0.035 × 0.045 × 0.051	3.95	6.2	—	—
adrcpt-For47	0.105 × 0.061 × 0.051	3.74	4.7	3.72	5.4
adrcpt-For48	0.105 × 0.039 × 0.064	—	—	3.80	5.7
adrcpt-For58	0.169 × 0.050 × 0.061	3.88	6.6	4.11	4.5
adrcpt-For59	0.042 × 0.024 × 0.025	3.25	9.2	3.16	8.3
adrcpt-For67	0.064 × 0.026 × 0.031	—	—	3.80	5.6

**Table 2:** Comparison of data-collection strategies and processing statistics for standard and adaptive beam-diameter protocols using a β_1_-andrenergic GPCR crystal Values in parentheses are for the outer shell.

Crystal	adrcpt-For42	adrcpt-For42
Beam diameter (µm)	50	30
Space group	*P*2_1_2_1_2_1_ [No. 19]	*P*2_1_2_1_2_1_ [No. 19]
Unit-cell parameters (Å, °)	*a* = 116.8, *b* = 121.1, *c* = 129.5, α = β = γ = 90	*a* = 116.5, *b* = 120.78, *c* = 128.7 α = β = γ = 90
Flux (photons s^−1^)	5.7 × 10^11^	2.2 × 10^11^
Rotation width (°)	0.15	0.05
Total oscillation range (°)	149.1	124.0
Total dose (MGy)	5.25	5.92
Detector resolution	4.05	3.9
Wilson *B* factor (Å^2^)	94.0	97.0
Resolution (Å)	48.2–3.95 (4.09–3.95)	48.7–3.53 (3.66–3.53)
Completeness (%)	98.2 (90.9)	81.5 (33.0)
Observed reflections	15940 (1413)	18785 (721)
Average multiplicity	4.1 (2.7)	4.4 (1.6)
〈*I*/σ(*I*)〉	4.3 (0.8)	10.6 (0.6)
*R*_meas_	0.23 (1.45)	0.1 (1.64)
*R*_merge_	0.18 (1.05)	0.077 (1.17)
CC_1/2_	0.99 (0.43)	1 (0.33)

**Table 3:** Pseudohelical data collection Comparison of data sets collected from a single position and from multiple positions for native and SAD strategies. Values in parentheses are for the outer shell.

	MXPressP		MXPressP_SAD	
	Single position	Multi-position	Single position	Multi-position
Protein	βPGM	βPGM	FAE	FAE
Crystal dimensions (*x* × *y* × *z*) (µm)	211 × 45 × 61	211 × 45 × 61	485 × 117 × 91	485 × 117 × 91
Space group	*P*2_1_2_1_2_1_	*P*2_1_2_1_2_1_	*P*4_1_2_1_2	*P*4_1_2_1_2
Unit-cell parameters (Å, °)	*a* = 52.7, *b* = 53.9, *c* = 81.5, α = β = γ = 90	*a* = 52.7, *b* = 53.9, *c* = 81.5, α = β = γ = 90	*a* = *b* = 111.4, *c* = 65.5, α = β = γ = 90	*a* = *b* = 111.4, *c* = 65.5, α = β = γ = 90
No. of positions	1	4	1	4
Beam diameter (µm)	30	30	100	100
Flux (photons s^−1^)	3.3 × 10^11^	3.3 × 10^11^	1.2 × 10^12^	1.2 × 10^12^
Transmission (%)	100	100	100	100
Dose per position (MGy)	8.23	5.8	16.53	5.85
Total dose (MGy)	8.23	23.2	16.53	23.40
Total exposure time (s)	115.8	326.8	275	390
Rotation width (°)	0.1	0.1	0.1	0.1
Total oscillation range (°)	125	120 [30 × 4]	360	360 [90 × 4]
Detector resolution (Å)	1.7	1.61	2.62	2.08
Resolution range (Å)	44.5–1.80	44.8–1.80	42.5–2.50	42.5–2.50
*R*_meas_	0.092 (0.94)	0.104 (0.74)	0.103 (0.974)	0.071 (0.522)
〈*I*/σ(*I*)〉	11.0 (1.6)	9.8 (1.3)	26.2 (5.2)	35.2 (8.6)
CC_1/2_	0.99 (0.57)	0.99 (0.77)	0.99 (0.93)	0.99 (0.98)
Multiplicity	4.2 (4.1)	4.3 (4.5)	24.7 (26.3)	26.2 (28.2)
Mid-slope of anomalous normal probability	—	—	1.49	1.80
Anomalous completeness (%)	—	—	100 (99.3)	100 (100)
CC_ano_	—	—	0.69 (0.07)	0.82 (0.13)
